# Targeting JARID1B’s demethylase activity blocks a subset of its functions in oral cancer

**DOI:** 10.18632/oncotarget.23739

**Published:** 2017-12-15

**Authors:** Nicole D. Facompre, Kayla M. Harmeyer, Varun Sahu, Phyllis A. Gimotty, Anil K. Rustgi, Hiroshi Nakagawa, Devraj Basu

**Affiliations:** ^1^ Department of Otorhinolaryngology, Head and Neck Surgery, The University of Pennsylvania, Philadelphia, PA, USA; ^2^ Department of Biostatistics Epidemiology and Informatics, The University of Pennsylvania, Philadelphia, PA, USA; ^3^ Department of Medicine, The University of Pennsylvania, Philadelphia, PA, USA; ^4^ Philadelphia VA Medical Center, Philadelphia, PA, USA; ^5^ The Wistar Institute, Philadelphia, PA, USA

**Keywords:** oral cancer, JARID1B, cancer stem cells, E-cadherin

## Abstract

Upregulation of the H3K4me3 demethylase JARID1B is linked to acquisition of aggressive, stem cell-like features by many cancer types. However, the utility of emerging JARID1 family inhibitors remains uncertain, in part because JARID1B’s functions in normal development and malignancy are diverse and highly context-specific. In this study, responses of oral squamous cell carcinomas (OSCCs) to catalytic inhibition of JARID1B were assessed using CPI-455, the first tool compound with true JARID1 family selectivity. CPI-455 attenuated clonal sphere and tumor formation by stem-like cells that highly express JARID1B while also depleting the CD44-positive and Aldefluor-high fractions conventionally used to designate OSCC stem cells. Silencing JARID1B abrogated CPI-455’s effects on sphere formation, supporting that the drug acted through this isoform. To further delineate CPI-455’s capacity to block JARID1B’s functions, its biologic effects were compared against those indicated by pathway analysis of the transcriptional profile produced by JARID1B knockdown. Downregulation of multiple gene sets related to stem cell function was consistent with the drug’s observed actions. However, strong E-Cadherin upregulation seen upon silencing JARID1B surprisingly could not be reproduced using CPI-455. Expressing a demethylase-inactive mutant of JARID1B demonstrated suppression of this transcript to be demethylase-independent, and the capacity of mutant JARID1B but not CPI-455 to modulate invasion provided a functional correlate of this finding. These results show that JARID1B catalytic inhibition effectively targets some stem cell-like features of malignancy but also reveal demethylase-independent actions refractory to inhibition. Future application of JARID1 inhibitors in combinatorial use for cancer therapy may be guided by these findings.

## INTRODUCTION

The epigenetic mechanisms that regulate embryonic and adult stem cell function are exploited during cancer progression. As a result, the dynamic histone modifications that underlie chromatin regulation in normal development and adult tissue homeostasis may also mediate related chromatin states that serve malignancy. Growing understanding of the enzymatic machinery of histone modification over the past decade has created interest in developing small molecule inhibitors of these enzymes for treating cancer. Currently, the only cancer drugs targeting histone modifiers in standard clinical use are histone deacetylase (HDAC) inhibitors, which have partial efficacy in certain malignancies [1].

In addition to HDACs, the methyl transferases and demethylases that act upon lysine and arginine residues in histones contribute to the epigenetic plasticity underlying malignant transformation and have thus become drug development targets [2]. Dysregulated expression of several histone demethylases is now implicated in cancer, with oncogenic or tumor suppressive roles having been attributed to all four members of the JARID1 (KDM5) family. This family is comprised of four isoforms (JARID1A, JARID1B, JARID1C, JARID1D), which are Fe^2+^ and 2-oxoglutarate (2-OG)-dependent oxidases with specificity for the di- and tri-methylated forms of lysine 4 in histone 3 (H3K4me2/3). JARID1 proteins are well known to repress transcription by removing the H3K4me3 mark at specific promoters [3–5]. However, they also participate in less studied gene-regulatory effects including transcriptional activation [6–9] and demethylase-independent actions [10] whose contributions to malignant progression are ill-defined.

Although JARID1C’s oncogenic versus tumor suppressive effects vary with cancer type [11–14], JARID1A and JARID1B primarily have tumor-promoting functions and are amplified in some malignancies [4, 9, 15, 16]. Such effects are best described for JARID1B, whose upregulation is implicated in many cancer types [17–25]. Even in the absence of generalized upregulation, high JARID1B levels in a small subset of tumor cells was shown to induce a slow-cycling state resistant to cytotoxic and targeted therapies in melanoma and OSCC cell lines [26–28]. This JARID1B^high^ state arose spontaneously and displayed stem cell-like molecular and functional properties that coincide with JARID1B’s regulation of the embryonic stem cell (ESC) state [7, 29, 30]. Our prior analyses of JARID1B^high^ OSCC cells also detected an epithelial-to-mesenchymal transition (EMT)-related gene expression profile [28] typically found in stem cell-like carcinoma cells. This profile included prominent downregulation of E-cadherin, a hallmark of EMT that is associated with JARID1B upregulation in cancer [25, 31] and has previously been attributed JARID1B’s demethylase-dependent repression of the miR-200 family [32]. Together these observations suggest that targeting JARID1B’s demethylase activity could address key epigenetic mechanisms underlying OSCC progression.

The contributions of JARID1B to cancer progression have led to the pursuit of JARID1 (KDM5) family-selective demethylase inhibitors, and the first small molecule inhibitor with high potency and true selectivity for the KDM5 family recently emerged. This prototype tool compound, CPI-455, has 200-fold selectivity for KDM5 over KDM4 demethylases, the most closely related family, and at least 500-fold selectivity over other KDM families [33]. In initial *in vitro* studies, CPI-455 sensitized cell lines of multiple cancer types to targeted inhibitors of their oncogenic drivers. Despite initial description of this tool drug, the effects of JARID1B demethylase inhibition upon tumor and host are largely unknown and hard to anticipate given the diverse, context-specific roles of this large multi-function protein.

Using CPI-455, we show for the first time that inhibiting JARID1B’s catalytic activity potently attenuates the stem cell-like molecular and functional features of cancer cells. However, the effects on E-cadherin expression and invasion seen by depleting JARID1B levels were surprisingly not recapitulated by CPI-455 treatment. Our findings provide a novel window into the biologic effects of JARID1 family-specific demethylase inhibition on the epigenetic plasticity that sustains malignant progression. Detection of demethylase-independent regulation of E-cadherin transcription also indicates that certain aspects of JARID1B function in cancer may prove refractory to catalytic inhibition.

## RESULTS

### CPI-455 attenuates the stem cell-like features of OSCCs

**Figure 1:**
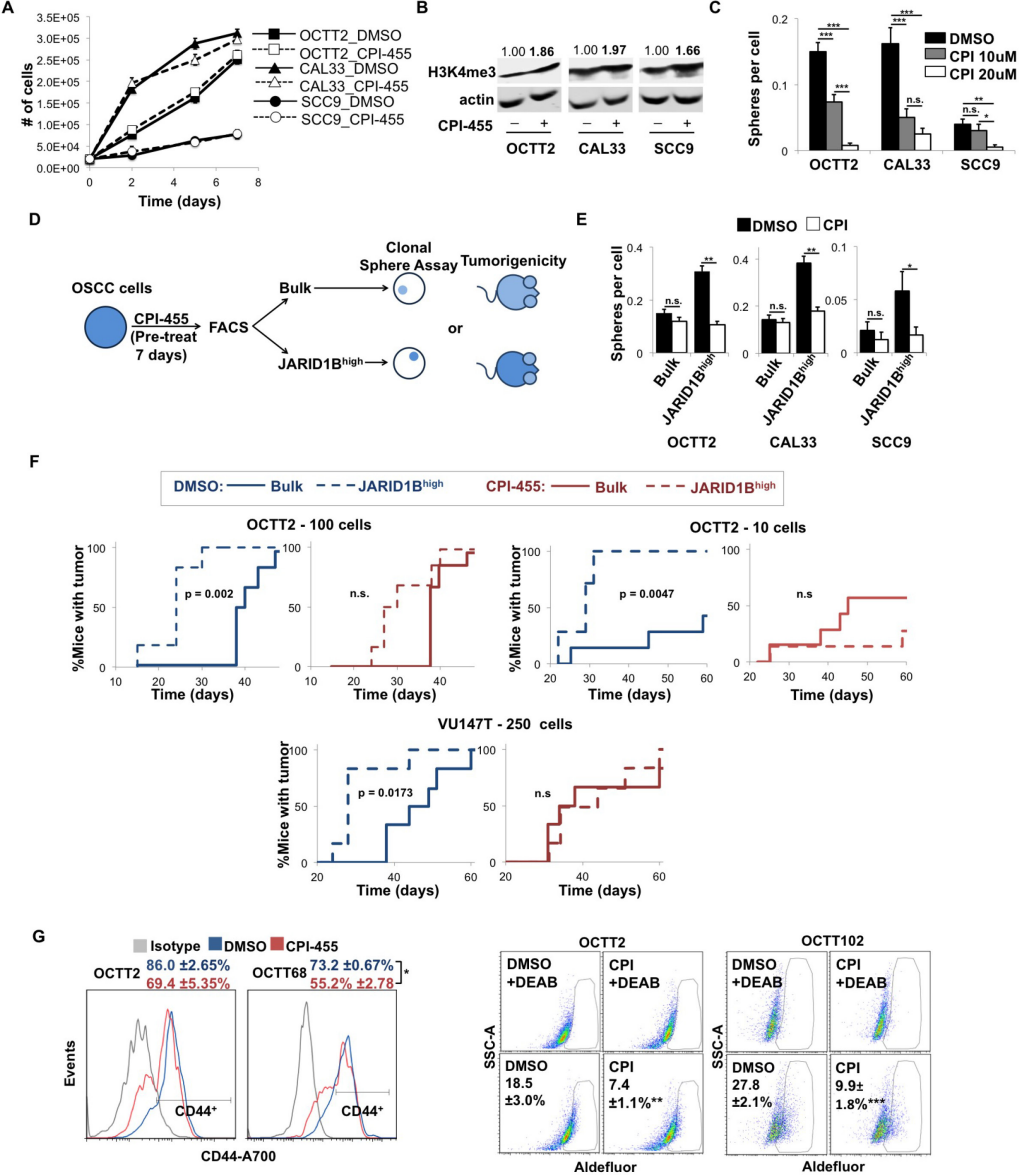
CPI-455 attenuates the stem cell-like features of OSCC cells (**A**) Cell growth over time of OSCC cells treated with CPI-455 (20 μM). (**B**) WB showing H3K4me3 levels in cell lines treated for 5 days with CPI-455 (20 μM). Values are H3K4me3 band densities normalized to actin. (**C**) Clonal sphere formation during continuous CPI-455 (CPI) treatment. ^*^p < 0.05, ^**^p < 0.001, ^***^p < 0.0001 (**D**) Schematic of CPI-455 pretreatment protocol. (**E**) Sphere formation by JARID1B^high^ vs. bulk OSCC cells after 7 days of pre-treatment with CPI-455 (20 μM). ^*^p < 0.05, ^**^p < 0.0001 (**F**) Xenograft formation by JARID1B^high^ vs. bulk OCTT2 (left: 100 cells/mouse, *n* = 6, middle: 10 cells/mouse, *n* = 7) or VU147T (right: 250 cells/mouse, *n* = 6) cells pre-treated for 7 days with CPI-455 (20 μM, red) or DMSO (blue). *p*-values are unadjusted. (**G**) Representative FC plots of CD44 (left) and Aldefluor (right) positive cells in primary cultures of PDXs after 5 days CPI-455 treatment (20 μM). Aldefluor positivity was defined as fluorescence above background in presence of the ALDH inhibitor DEAB. ^*^p < 0.05, ^**^p < 0.025, ^***^p < 0.01.

Recent availability of CPI-455 provided the first opportunity to define effects of pharmacologic inhibition of JARID1 demethylases upon the stem cell-like traits conferred by high JARID1B levels. Previously reported properties of CPI-455 were first validated using OSCC cell lines. As in other cancer types [33], doses up to 25 μM did not impact cell proliferation (Figure 1A). The reported time frame of optimal target inhibition was confirmed in our system based on an increase in global H3K4me3 levels after 5 days of exposure to 20 μM CPI-455 (Figure 1B, Supplementary Figure 1A). Despite not impacting cell viability or apoptosis in standard culture (Supplementary Figure 1B-1C), CPI-455 potently inhibited clonal sphere formation in serum-free media in a dose-dependent manner (Figure 1C). This activity was further characterized by examining the drug’s effects on the fraction of OSCC cells in which JARID1B is spontaneously upregulated. As reported by us previously [28], these JARID1B^high^ cells have markedly enhanced clonal sphere and tumor-forming capacity and can be isolated using the promoter-based lentiviral reporter J1BpromEGFP. Because the undefined *in vivo* pharmacology for CPI-455 prevented continuous treatment in a xenograft model, a protocol was developed where cell lines were instead pretreated *in vitro* with 20 μM CPI-455 for 7 days before purifying JARID1B^high^ cells for functional assessments performed in the absence of drug (Figure 1D). This protocol also allowed for evaluation of the effects of the drug on the initiation of sphere- or tumor-formation by the JARID1B^high^ fraction independent of effects on progression. CPI-455 treatment did not alter the size of the JARID1B^high^ fraction (Supplementary Figure 1D) but abrogated its enhanced sphere-forming ability while not impacting the lower efficiency of sphere formation by bulk cells (Figure 1E). Retention of CPI-455′s effects in the absence of continuous treatment underscored its activity against a stem cell-like phenotype. Importantly, *in vitro* pretreatment alone also impaired the more efficient initiation of xenograft tumors by JARID1B^high^ cells compared with the bulk population using two OSCC cell lines at limiting doses (Figure 1F). CPI-455 pretreatment also significantly increased the time-to-tumor in the JARID1B^high^ but not bulk groups of OCTT2 xenograft mice (Supplementary Figure 2).

Analysis of the drug’s effects was extended to cell fractions with either high cell surface CD44 or aldehyde dehydrogenase (ALDH) activity, which are widely used markers of stem-like function in OSCC [34, 35] and decrease upon JARID1B knockdown [28]. Because intra-tumor heterogeneity in ALDH activity and CD44 is often depleted in established cancer cell lines, three OSCC patient-derived xenografts (PDXs) were used instead to establish a feeder-assisted primary culture system. Growing tumor colonies were readily distinguished from mouse stroma and feeder fibroblasts by morphology and anti-human HLA staining (Supplementary Figure 3). Two of the PDX primary cultures displayed discrete CD44^high^ and CD44^low^ subsets and two contained a minority Aldefluor-positive fraction. In those cases, CPI-455 depleted the CD44^high^ fraction (Figure 1G, left) and decreased Aldefluor-positive cells (Figure 1G, right). Together, these data demonstrate that catalytic inhibition of JARID1 demethylases depletes stem-like cells in OSCC and attenuates the tumor-initiating capacity attributed to them.

### CPI-455 alters stem cell-like features in OSCC primarily by inhibiting the JARID1B isoform

**Figure 2:**
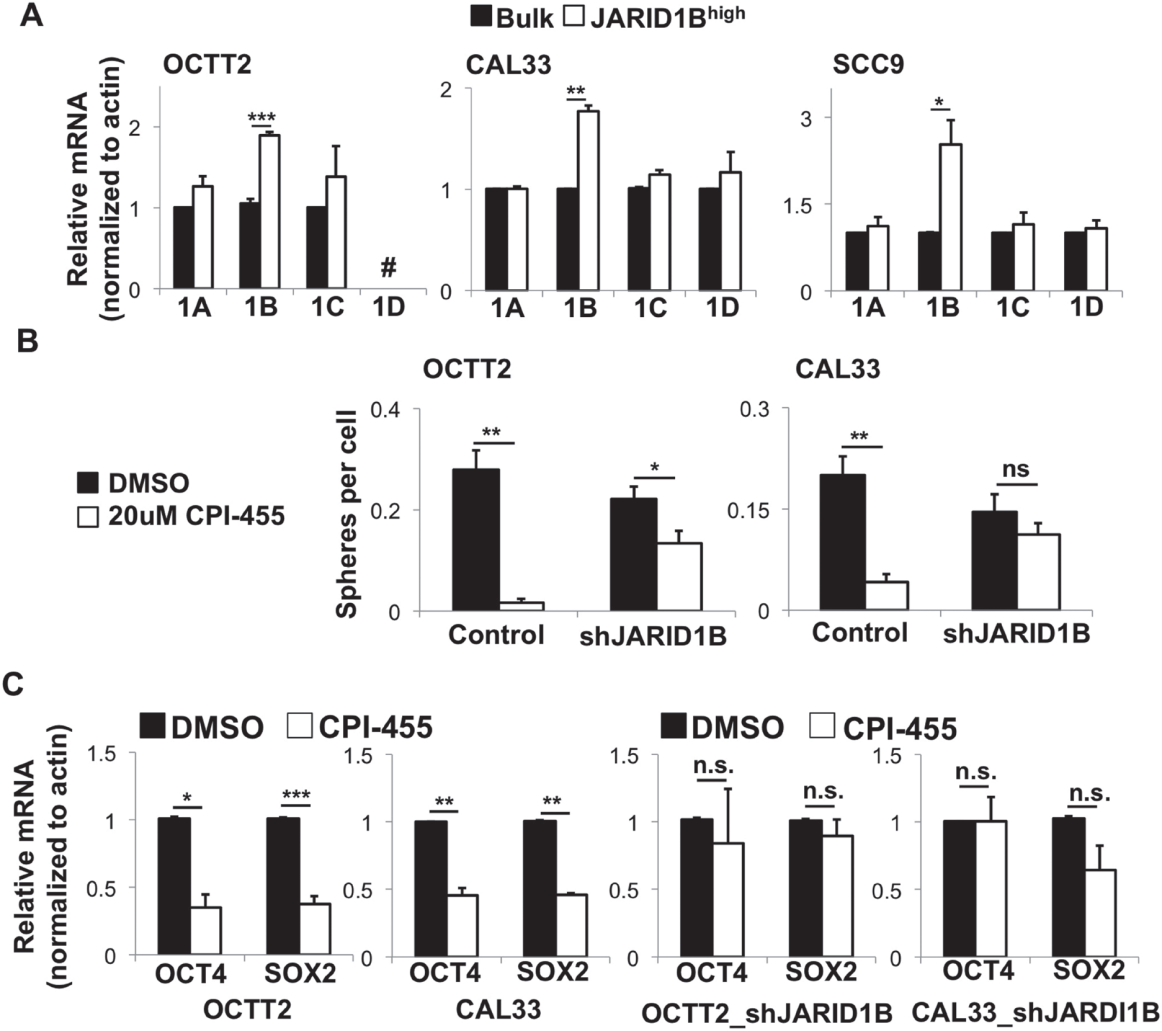
CPI-455 alters stem-like features in OSCC primarily by inhibiting the JARID1B isoform (**A**) Expression of JARID1 family members by QRT-PCR in bulk vs. JARID1B^high^ cells from OSCC lines. # = not done because JARID1D is Y-linked, and cell line is of female origin. ^*^p < 0.05, ^**^p < 0.005, ^***^p < 0.0005. (**B**) Sphere formation during continuous CPI-455 (20 μM) treatment after stable JARID1B silencing. ^*^p < 0.025, ^**^p < 0.0001. (**C**) OCT4 and SOX2 mRNA expression by QRT-PCR in shScramble control (left) and shJARID1B (right) OSCC lines treated for 5 days with CPI-455 (20 μM). ^*^p < 0.05, ^**^p < 0.025, ^***^p < 0.01.

Although JARID1B is the JARID1 isoform most strongly linked to cancer progression, CPI-455 inhibits all four family members with similar potency [33]. Thus, inhibiting the other three isoforms might also have determined CPI-455′s effects on stem cell-like gene expression and function. However, the A, C, and D isoforms were found not to be upregulated in unison with JARID1B in the JARID1B^high^ fraction (Figure 2A). Furthermore, shRNA-mediated depletion of JARID1B desensitized OSCC cell lines to the strong effect of CPI-455 on clonal sphere formation shown in Figure 1 (Figure 2B). Despite this effect, stable silencing of JARID1B alone had only a modest effect on sphere formation. This result may be explained by incomplete knockdown of JARID1B levels (Supplementary Figure 4A) but is also likely contributed to by long term epigenetic compensation upon stable silencing of the chromatin regulator. Effects of CPI-455 on stem cell-related gene transcription were then evaluated at the level of OCT4, a transcript positively regulated by both JARID1A and JARID1B [7, 36–38], and SOX2, another mediator of pluripotency often upregulated in stem-like OSCC cells [39, 40]. CPI-455 repressed OCT4 and SOX2 in OSCC cell lines (Figure 2C, Supplementary Figure 4B). This effect was abrogated upon JARID1B silencing (Figure 2C), which alone decreased OCT4 and SOX transcript levels (Figure 2C, Supplementary Figure 4C). Collectively, these findings support our hypothesis that CPI-455′s effects on stem cell-like molecular and functional traits of OSCCs are mediated predominantly through the JARID1B isoform.

### E-cadherin repression is a prominent JARID1B-mediated effect in OSCC

**Figure 3:**
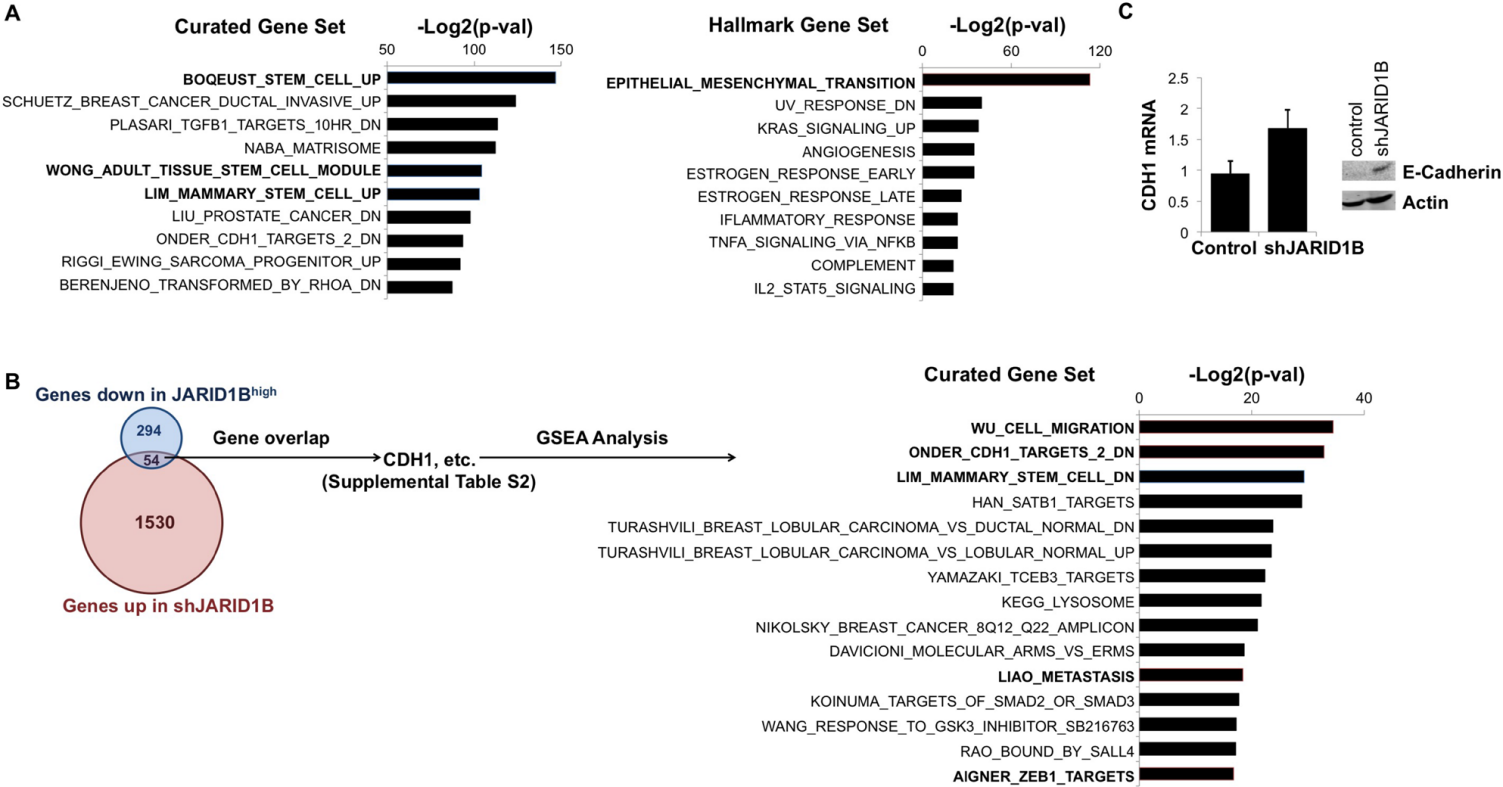
JARID1B silencing suppresses stem cell-related gene expression and upregulates E-cadherin (**A**) Most significantly downregulated gene sets in the MSigDB Curated (C2:4,725 sets) database (left) and Hallmark (H:50 sets) database (right) after silencing JARID1B in OCTT2 cells. (**B**) Overlap among genes up-regulated in shJARID1B OCTT2 cells and down-regulated in JARID1B^high^ OCTT2 cells (left). Most enriched C2:Curated gene sets among the 54 genes both up-regulated in shJARID1B cells and down-regulated in JARID1B^high^ cells (right). (**C**) CDH1 (E-cadherin) mRNA and protein expression by QRT-PCR and WB, respectively, in OCTT2 cells after stable JARID1B silencing.

We attempted to predict additional molecular effects that result from targeting JARID1B with CPI-455 using mRNA sequencing data that compares OCTT2 cells in the presence or absence of shRNA-mediated JARID1B silencing. Gene set enrichment analysis (GSEA) was performed on the ~3000 genes differentially expressed after JARID1B knockdown (Supplementary Table 1) using the gene sets selected from the Broad Institute Molecular Signature Database (MSigDb). The H:Hallmark gene collection was analyzed to detect relationships to well-defined cell states, and C2:Curated was chosen as a diverse collection that includes some stem cell-related phenotypes. As anticipated, the Curated pathways most downregulated after silencing JARID1B included the stem cell-related processes (Figure 3A, left). Similarly, the single most downregulated process in the Hallmark gene collection was EMT (Figure 3A, right), a phenomenon closely linked to acquisition of stem cell-like traits in carcinomas [41].

The genes upregulated upon silencing JARID1B were regarded as possible direct JARID1B targets based on the JARID1 family’s most studied gene-regulatory mechanism being transcriptional repression. This gene list was narrowed by examining overlap with our published profile of transcripts decreased in JARID1B^high^ cells relative to the bulk pool [28] in order to identify genes potentially repressed by JARID1B in context of malignancy (Figure 3B, left). The overlap consisted of 54 genes (Supplementary Table 2), for which GSEA again showed links to stem cell function and EMT-associated processes like upregulation of Zeb1 target genes, cell migration, and metastasis (Figure 3B, right). The overlap also confirmed strong association of JARID1B expression with E-cadherin downregulation, a hallmark of EMT, which was consistent with enrichment of genes known to be downregulated following E-cadherin loss in cancer (ONDER_CDH1_TARGETS_2_DN). The effect of JARID1B silencing on E-cadherin expression was subsequently demonstrated by QRT-PCR and western blot (WB) in OCTT2 cells (Figure 3C). These results demonstrate JARID1B’s contribution to the stemness and EMT-related transcriptional profiles found in OSCC and suggested JARID1B-mediated E-cadherin repression to be one possible mediator of CPI-455′s effects.

### JARID1B-mediated E-cadherin repression in OSCC is demethylase-independent and insensitive to CPI-455

**Figure 4:**
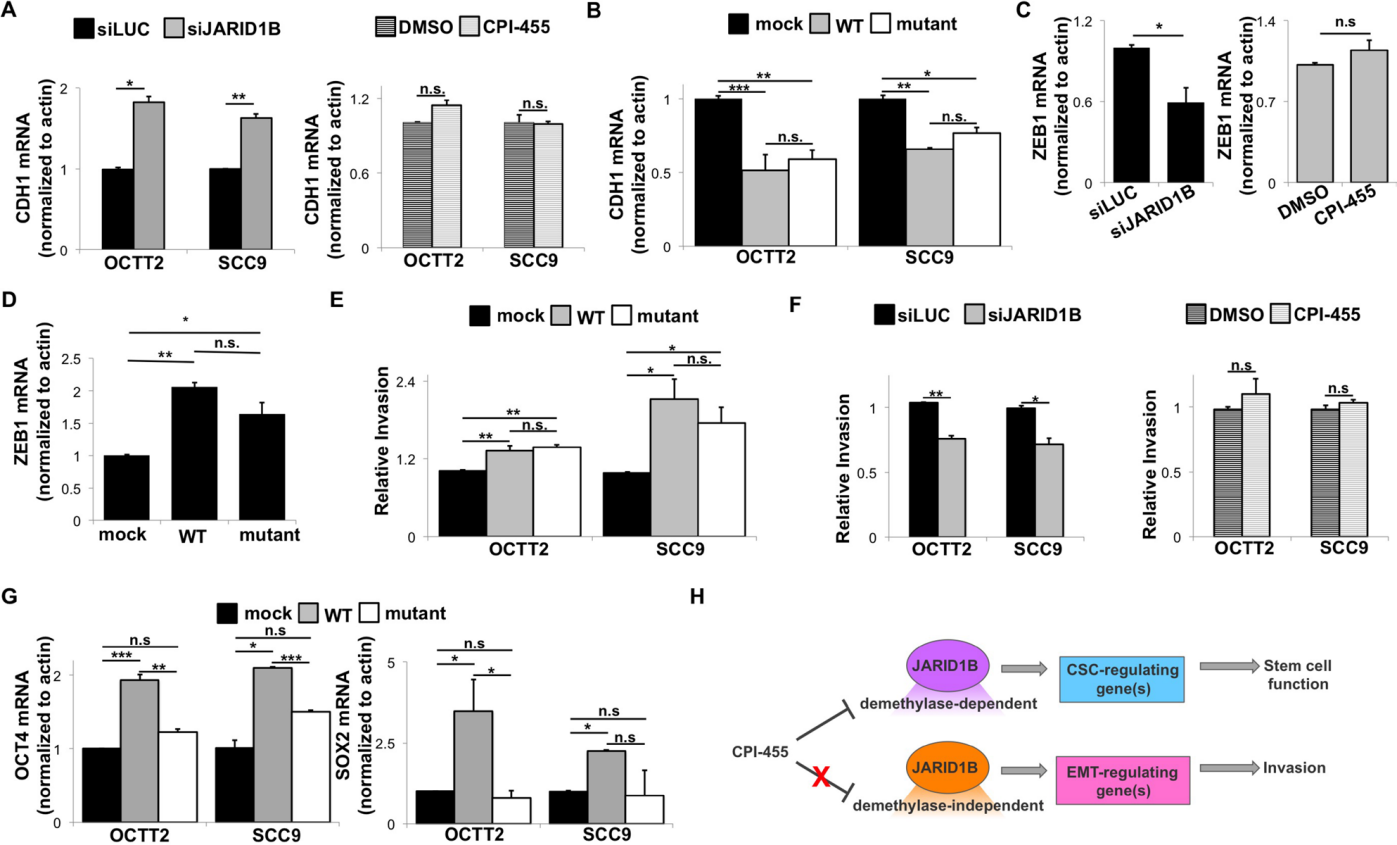
JARID1B-mediated E-cadherin repression in OSCC can be demethylase-independent (**A**) E-cadherin (CDH1) mRNA by QRT-PCR after transient siRNA knockdown of JARID1B (left) or 5-day treatment with CPI-455 (20 μM, right). ^*^p < 0.025, ^**^p < 0.01 (**B**) E-cadherin (CDH1) mRNA by QRT-PCR after transiently overexpressing WT or mutant (H499Y) JARID1B. ^*^p < 0.025, ^**^p < 0.01, ^***^p < 0.005 (**C**) ZEB1 mRNA expression in OCTT2 cells by QRT-PCR after transient siRNA knockdown of JARID1B (left) or 5-day treatment with CPI-455 (20 μM, right). ^*^p < 0.05 (**D**) ZEB1 mRNA in OCTT2 cells by QRT-PCR after transiently overexpressing WT or mutant (H499Y) JARID1B. ^*^p < 0.025, ^**^p < 0.0001 (**E**) Transwell collagen invasion relative to indicated controls after transiently overexpressing WT or mutant (H499Y) JARID1B. ^*^p < 0.05, ^**^p < 0.0025 (**F**) Transwell collagen invasion after transient siRNA knockdown of JARID1B (left) or 5-day treatment with CPI-455 (20 μM, right). ^*^p < 0.025, ^**^p < 0.01 (**G**) OCT4 and SOX2 mRNA expression by QRT-PCR after transiently overexpressing WT or mutant (H499Y) JARID1B. ^*^p < 0.05, ^**^p < 0.025, ^***^p < 0.01 (**H**) Model proposing segregation of demethylase-dependent vs independent actions of JARID1B that dictate the response to catalytic inhibition.

Depletion of JARID1B levels using siRNA (Supplementary Figure 5A) further validated its inverse effect on E-Cadherin transcription in OSCC cell lines (Figure 4A left). However, CPI-455 treatment surprisingly failed to alter E-cadherin mRNA levels (Figure 4A right), leading to assessment of whether JARID1B’s regulation of E-cadherin requires its H3K4 demethylase activity. Overexpression of the catalytically inactive H499Y mutant form of JARID1B [4] did not impact global H3K4me3 levels (Supplementary Figure 5B–5C) but reduced E-cadherin transcription equally as effectively as wildtype (WT) JARID1B (Figure 4B). Similarly, expression of the negative E-Cadherin regulator Zeb1 was not impacted by CPI-455 (Figure 4C) and overexpression of either WT or mutant JARID1B increased Zeb1 levels (Figure 4D). A functional correlate of this demethylase-independent regulation of E-cadherin was provided by finding that overexpression of WT or the H499Y mutant JARID1B enhanced invasion equivalently in a collagen transwell assay (Figure 4E). Accordingly, downregulating JARID1B levels reduced invasion, whereas treatment with the catalytic inhibitor failed to do so (Figure 4F). By contrast, OCT4 and SOX2 levels were sensitive to CPI-455 (Figure 1) and accordingly increased only upon overexpression of WT JARID1B and not the H499Y mutant (Figure 4G). These results reveal that JARID1B can suppress E-cadherin levels and promote OSCC invasion by demethylase-independent mechanisms that are refractory to CPI-455. At the same time they confirm the demethylase dependence and drug sensitivity of other JARID1B-mediated effects that impact the OSCC stem cell phenotype (Figure 4H).

## DISCUSSION

This study of OSCCs found that upregulation of JARID1B mediates a stemness- and EMT-related gene expression profile that includes strong downregulation of E-cadherin. CPI-455, a uniquely potent and selective inhibitor of JARID1 family demethylases, acted predominantly through the JARID1B isoform to attenuate stem cell-like molecular and functional traits in OSCCs. However, JARID1B-mediated repression of E-cadherin and promotion of invasion proved independent of demethylase activity and thus insensitive to CPI-455. Our findings reveal the capacity of emerging histone demethylase inhibitors to target discrete epigenetic determinants of malignant cell states, suggesting their future utility in combination therapies. At the same time, they highlight unexplored demethylase-independent roles of JARID1 proteins in cancer that are refractory to catalytic inhibition.

The JARID1 family’s contributions to normal development provide insight into its regulation of stem-like cell phenotypes in malignancy and our novel findings on the effects of small molecule inhibition in OSCC. The peak expression of JARID1A and JARID1B in early embryos [29] coincides with their reported contributions to regulation of pluripotency [30, 42]. JARID1B and JARID1C have essential functions in early development based on deletion of either isoform causing aberrant neural lineage specification [3, 42] and embryonic lethality [43, 44], whereas JARID1A knockouts are viable and fertile [43]. In later development and tissue homeostasis, JARID1 proteins are implicated in multiple cell state transitions whose attributes might also be exploited by cancers. These roles include myogenic differentiation of embryonic fibroblasts [45], postnatal mammary development [9, 43], hematopoietic stem cell differentiation [46], adult neural stem cell maintenance [47], and angiogenesis [48].

Among the four JARID1 family members, JARID1B’s strongest link to cancer progression corresponds to its pivotal contribution here to the effects of inhibiting all isoforms with CPI-455. JARID1B’s tumor-promoting function is perhaps best characterized in breast cancer, where it is a *bona fide* oncogene that is amplified and overexpressed in some hormone receptor-positive tumors and drives a luminal-type gene expression profile [4, 9]. It is similarly upregulated in numerous other cancer types including gastric [17], prostate [18], bladder [19], ovarian [20], esophageal [21], lung [22], lymphoma [23, 24], and neuroblastoma [25]. Recent association between JARID1B expression and stem cell-like de-differentiation in multiple squamous epithelial malignancies [16, 28, 37, 49] is consistent with the shifts in gene expression observed upon silencing it in OSCC in this study. Upregulating JARID1A can also be advantageous in certain cancers including OSCC [15, 16], and JARID1C expression has similarly been associated with poor prognosis in breast and prostate cancers [11, 12]. These partly overlapping roles for JARID1 family members may confer advantages to isoform-nonselective inhibitors like CPI-455. At the same time, inactivation or loss of JARID1C and JARID1D in a few cancer types reveals potential tumor suppressive roles [13, 14, 50, 51] that may make targeting all isoforms counterproductive in certain contexts.

Until recently, specific targeting of JARID1 demethylases was impaired by close structural conservation of the JARID1 catalytic core across most histone lysine demethylases. Thus early inhibitors of the JARID1 family were simple Fe^2+^ chelators or competitive 2-OG analogs that impede a catalytic mechanism shared by many demethylases [52–54]. However, the KDM5 family is distinguished from multiple similar demethylase families by division of its catalytic domain by a DNA-binding ARID domain and a PHD finger into two components, JmjN and JmjC, which are both required for activity. In-depth structural analyses have sought to improve potency and selectivity of inhibitors based on distinct amino acid side chains and conformational plasticity in JARID1 active sites [55, 56]. Although the CPI-455 tool compound is presently the best validated result of this effort [33], 1,7-naphthyridones are another recently defined small molecule class with possible JARID1 family specificity [57]. Other lead compounds are reported to have selectivity for JARID1A [58, 59], which makes isoform-specific inhibition appear feasible.

The biologic effects of emerging inhibitors are largely unknown and difficult to anticipate given the complex and highly context-dependent mechanisms of gene regulation by these large multi-function proteins. Most mechanistic studies of the family’s oncogenic functions have focused on transcriptional repression of specific genes by removing activating H3K4me2/3 marks at proximal promoters. In this role, JARID1B enhances oncogenic PI3-kinase signaling by repressing PTEN [31] and promotes cell cycle progression by silencing transcription of p27^kip1^ and other cdk inhibitors [60–62]. In general, such functions are mediated by incorporation of JARID1 demethylases into larger repressive complexes containing other chromatin regulators including histone methyltransferases, other histone demethylases, and HDACs [63–66].

Removal of H3K4me3 more widely across the genome by JARID1 proteins can have gene-regulatory effects other than transcriptional repression and may explain the actions of JARID1B observed here. For instance, JARID1B-mediated H3K4 demethylation within bodies of genes can promote their expression by suppressing cryptic intragenic transcription [6]. Similarly, H3K4me3/2 removal by JARID1B and JARID1C just outside the boundaries of enhancers can increase expression by augmenting enhancer function [7, 8]. Such JARID1B-mediated effects have been linked to OCT4 promoter activity in ESCs [7] and thus are possible mechanisms for the positive regulation of OCT4 and other stem cell-related genes observed here.

The inhibitory effects of CPI-455 treatment seen here on the sphere and tumor forming capacities of OSCC cells are strongest in the JARID1B^high^ subfraction and attenuated upon knockdown of JARID1B, further linking the actions of the drug to this specific isoform. However, shRNA-mediated depletion of JARID1B shows limited effects on the bulk population of OSCC cells. This modest effect could be explained by either the incomplete knockdown achieved by shRNA (Supplementary Figure 2) or long term epigenetic compensation due to reduced JARID1B levels. Such compensation would account for the diminished effect of CPI-455 in presence of JARID1B shRNA in Figure 2B. Importantly, we previously showed that silencing JARID1B has far greater effects on the small, treatment resistant JARID1B^high^ cell fraction where its levels are spontaneously elevated [28]. The enhanced sphere and tumor formation capacity of these JARID1B^high^ cells is abrogated by shRNA, and the comparable effect of CPI-455 in this study (Figure 1) supports the relevance of JARID1B as a target for addressing heterogeneity in the tumor cell pool.

An even wider range of gene regulatory effects for the JARID1 family is revealed by our finding that JARID1B’s does not require its catalytic activity to repress E-cadherin and promote invasion. This result offers new insight into the reciprocal relationship between E-cadherin and JARID1B expression observed in multiple cancer types [25, 31, 32]. JARID1B’s EMT-promoting effects were previously proposed to occur downstream of its activation of PI3-kinase signaling via direct repression of the PTEN promoter [31]. Effects of JARID1B on E-cadherin levels have also been attributed to JARID1B directly reducing expression the miR-200 family of microRNAs that negatively regulate the Zeb1 and Zeb2 transcriptional repressors of E-cadherin [32]. Remarkably, such demethylase-dependent effects on E-cadherin did not predominate in our system. JARID1B’s demethylase-independent functions are largely unexplored in cancer but are predictable from studies of Lid, the single JARID1 family homologue in *D. melanogaster.* Lid’s catalytically inactive form rescues the embryonic lethality of its deletion mutant [67] and promotes transcription by diverse mechanisms [68–70]. Although studies of demethylase-independent actions in mammalian cells are scant, one such function is reported for JARID1A in regulation of circadian rhythm. In this role, it acts in a complex with the transcription factors CLOCK and BMAL1 to increase Per2 promoter activity in a cyclic manner [10]. A direct action of JARID1B may occur at the FOXA1 promoter, where it recruited GATA3 to support mammary morphogenesis [9], although the demethylase independence of this effect was not determined.

Emerging roles for JARID1B and other JARID1 family members in contextual regulation of cell cycle progression raises questions regarding the effects of demethylase inhibition on the cycling status of OSCC cells. Both JARID1A and JARID1B can promote G_1_-S progression but also maintain G_0_/G_1_ arrest in fibroblasts through suppression of pRb target genes [71]. They have also been shown to have the ability to repress multiple cdk inhibitors in various contexts [15, 60–62]. Notably, high levels of JARID1B are a hallmark of a slow-cycling, drug-resistant subpopulation in melanoma and OSCC [26–27]. Further studies are needed to pursue the effects of CPI-455 on the turnover rates of OSCC cells and, specifically, the JARID1B^high^ fraction.

In sum, our results indicate that JARID1B upregulation exerts complex tumor-promoting effects in OSCC through both demethylase-dependent and independent mechanisms. Thus, determining which of its contributions to cancer progression require catalytic activity is needed to clarify the features of cancers that are readily targetable using emerging JARID1 family inhibitors. Doing so can facilitate future use of these agents in combination therapies that address the epigenetic plasticity underlying escape of cancer cells from current drugs.

## MATERIALS AND METHODS

### Cell lines and PDX primary cultures

SCC9 cells were obtained from the ATCC. OCTT2 cells are previously described [72]. CAL33 cells were a gift from Dr. Jennifer Grandis, University of California San Francisco. VU147T cells were gifted by Dr. Hans Joenje, VU Medical Center, Netherlands. Cell lines were authenticated using the AmpFISTR^®^ Identifier PCR Amplification kit (Applied Biosystems, Foster City, CA) and maintained in 1:1 DMEM/F12 media with 400 ng/ml hydrocortisone, 10% FBS, and 50 μg/ml gentamycin. We previously reported the generation and maintenance of the PDXs used here [73]. Three HPV- PDX tumors, OCTT2 (derived from the same primary tumor as the OCTT2 cell line), OCTT68, and OCTT102, were dissociated as described by us [28] into single cell suspensions and plated onto ~80% confluent, irradiated (30 Gy) NIH/3T3 cells. Cultures were grown in 1:1 DMEM/F12 with 10% FBS, 400 ng/ml hydrocortisone, 50 μg/ml gentamycin, 0.5 ng/ml recombinant human EGF, 10 ng/ml cholera toxin, and 5μM ROCK inhibitor Y-27632.

### Vectors and RNAi

Specific silencing of JARID1B using lentiviral vector pLKO-shJARID1B (shJARID1B) is previously described [26], and transient JARID1B overexpression was performed using pBIND-RBP2H1, a gift from Dr. Alexander Roesch, University Hospital Essen, Germany [74]. The H499Y mutation was introduced into pBIND-RBP2H1 through site directed mutagenesis using these primers:

forward: 5′-GAATGTGCTTTTCTTCATTCTGTT GGTACATTGAAGACCACTGGAGCTATTCAATT AAC-3′

reverse: 5′- GTTAATTGAATAGCTCCAGTGGT CTTCAATGTACCAACAGAATGAAGAAAAGCACA TTC-3′

SiRNA experiments used a Mission^Ⓡ^ esiRNA (Sigma, St. Louis, MO) with previously demonstrated specificity for JARID1B [37] and a luciferase specific control (siLuc). Transient transfections were performed in antibiotic-free media using Lipofectamine LTX&Plus reagent per manufacturer instructions (Invitrogen, Carlsbad, CA).

### Drug treatment and target inhibition

Target inhibition using CPI-455 (Axom Medchem, Reston, VA) was monitored by western blotting for global H3K4 trimethylation. Equal numbers of CPI-455- or vehicle-treated cells were lysed in Laemmli buffer. Protein lysates were separated on 10% ECL gels (GE, Pittsburgh, PA), and transferred to nitrocellulose membranes using the Trans-Blot^®^ System (Bio-Rad, Hercules, CA). Anti-H3K4me3 (Active Motif, Carlsbad, CA) diluted 1:1000 was incubated overnight at 4°C. After washing, *anti-Rabbit* IgG-DyLight was added. Blots were analyzed using the LI-COR Odyssey System (LI-COR, Lincoln, NE).

### Tumorigenicity

Tumorigenicity was determined as described previously [28] using non-obese diabetic/severe combined immunodeficient/interleukin-2 receptor g-chain-deficient (NSG) mice under Wistar Institute IACUC protocol 112652. Cell lines in 100 μl Matrigel were injected subcutaneously into the flank of NSG mice. Tumor incidence and latency were monitored over 60 days post-injection.

### Flow cytometry (FC) and JARID1B*^high^* cell isolation

Tumor cells from PDX primary cultures were distinguished from mouse stromal cells by pre-treating with anti-mouse CD16/32-Fc-Block (BD) before staining with mouse monoclonal anti-human HLA-ABC (eBiosciences, San Diego, CA). Surface CD44 was measured using anti-CD44-Alexafluor700 (eBiosciences). ALDH activity was determined using Aldefluor assays per manufacturer instructions (Stemcell, Vancouver, Canada). Propidium iodide was used for dead cell exclusion throughout. FC was performed on an LSRII instrument (BD, San Jose, CA). JARID1B^high^ cells were isolated based on 5% highest EGFP intensity in J1BpromEGFP-expressing cells lines by fluorescence-activated cell sorting using an AstriosEQ (Beckman-Coulter, Miami, FL). Elevated JARID1B mRNA and protein levels in this cell fraction were confirmed as previously described [28].

### Cell viability, proliferation, apoptosis, and invasion assays

Cell viability was assessed using the CellTiter 96^®^ AQ_ueous_ One Solution Cell (Promega, Madison, WI) per manufacturer’s instructions. Cell proliferation was monitored by trypan exclusion assay 2, 5, and 7 days post-treatment. Apoptosis was measured using the PE Annexin V Apoptosis Detection Kit (Biolegend, San Diego, CA) per manufacturer’s instructions. Invasion was assessed using the Cultrex^®^ Collagen I Cell Invasion Assay (Trevigen, Gaithersburg, MD) per manufacturer’s instructions using 20% FBS in the bottom chamber as a chemo-attractant. After 48 hours, Calcein-AM was added to the bottom chamber, where invasive cells were quantified using an FLx800 plate reader (Biotek, Winooski, VT).

### Sphere formation

10 (OCTT2, CAL33) or 20 (SCC9) cells/well were cultured in serum-free complete MEGM (Lonza, Basel, Switzerland) on ultralow attachment 96-well plates (Corning, Corning, NY) as detailed by us previously [28]. Spheres were counted 14 days after plating.

### Real-time reverse transcription PCR and RNA-Seq

QRT-PCR was performed as previously described [28]. Primers sequences are as follows:

Actin forward 5′-CGCGAGAAGATGACCCAG AT-3′, reverse 5′-GATAGCACAGCCTGGATAGCAA C-3′;

E-Cadherin forward 5′-TGCCCAGAAAATGAAAA AGG-3′, reverse 5′-GTGTATGTGGCAATGCGTTC-3′; JARID1A forward 5′-GCTTGGCAATGGGAACAA AA-3′, reverse 5′-CCGTTGTCTCATTTGCATGTTAA-3′; JARID1B forward 5′-AACAACATGCCAGTGATGG A-3′, reverse 5′-TACCAGGTTTTTGGCTCACC-3′;

JARID1C forward 5′-GGCTTAGAGAATGGA GAC-3′, reverse 5′-TCAGGCAGTTCCAACACAG-3′;

JARID1D forward 5′-AGCCAACCATGTGCAATG TA-3′, reverse 5′-GGCTCTGGATCAGGCTGTAG-3′;

OCT4 forward 5′-GTTGGAGGGAAGGTGAAG TT-3′, reverse 5′-CTGTGTCCCAGGCTTCTTTAT-3′; SOX2 forward 5′-GCCGAGTGGAAACTTTTGTCG-3′, reverse 5′-GCAGCGTGTACTTATCCTTCTT-3′ Total RNA was collected from OCTT2 cells stably transfected with either shJARID1B or an shScramble control for mRNA sequencing RNA-Seq, which was performed as for JARID1B^high^ vs. bulk cells previously [28]. Briefly, multiplexed Illumina libraries were prepared with the Illumina stranded mRNA kit. Libraries were pooled and sequenced to 100 bp from one end of the insert. Resulting reads were aligned against the human genome (hg19) using RUM (version 2.0.4). Differentially-expressed genes were defined using EdgeR and had an FDR > 10%. The data sets are available from the corresponding author upon request. MSigDb (http://software.broadinstitute.org/gsea/msigdb/index.jsp) was used to assess overlaps with existing transcriptional profiles.

### Statistics

Data represent at least three experimental replicates and are expressed as mean ± standard error. ANOVA or *t*-tests were used to evaluate significant differences among means. Tukey’s procedure was used to evaluate pairwise differences when ANOVAs were significant. Kaplan-Meier and exact log-rank tests defined differences among cumulative distributions for tumor latencies.

## SUPPLEMENTARY MATERIALS FIGURES AND TABLES



## Abbreviations

2-OG: 2-oxoglutarate
CSC: cancer stem cell
EMT: epithelial-to-mesenchymal transition
ESC: embryonic stem cell
FC: flow cytometry
HDAC: histone deacetylase
OSCC: oral squamous cell carcinoma
PDX: patient-derived xenograft
WB: western blot
WT: wildtype
