# Supplementary material for: Targeting JARID1B’s demethylase activity blocks a subset of its functions in oral cancer
Source: Oncotarget. 2017 Dec 15;9(10):8985–98. doi: 10.18632/oncotarget.23739 (PMC5823649; doi:10.18632/oncotarget.23739)
Supplement: Supplementary file 1 [file oncotarget-09-8985-s001.pdf]

Targeting JARID1B's demethylase activity blocks a subset of its functions in oral cancer

SUPPLEMENTARY MATERIALS

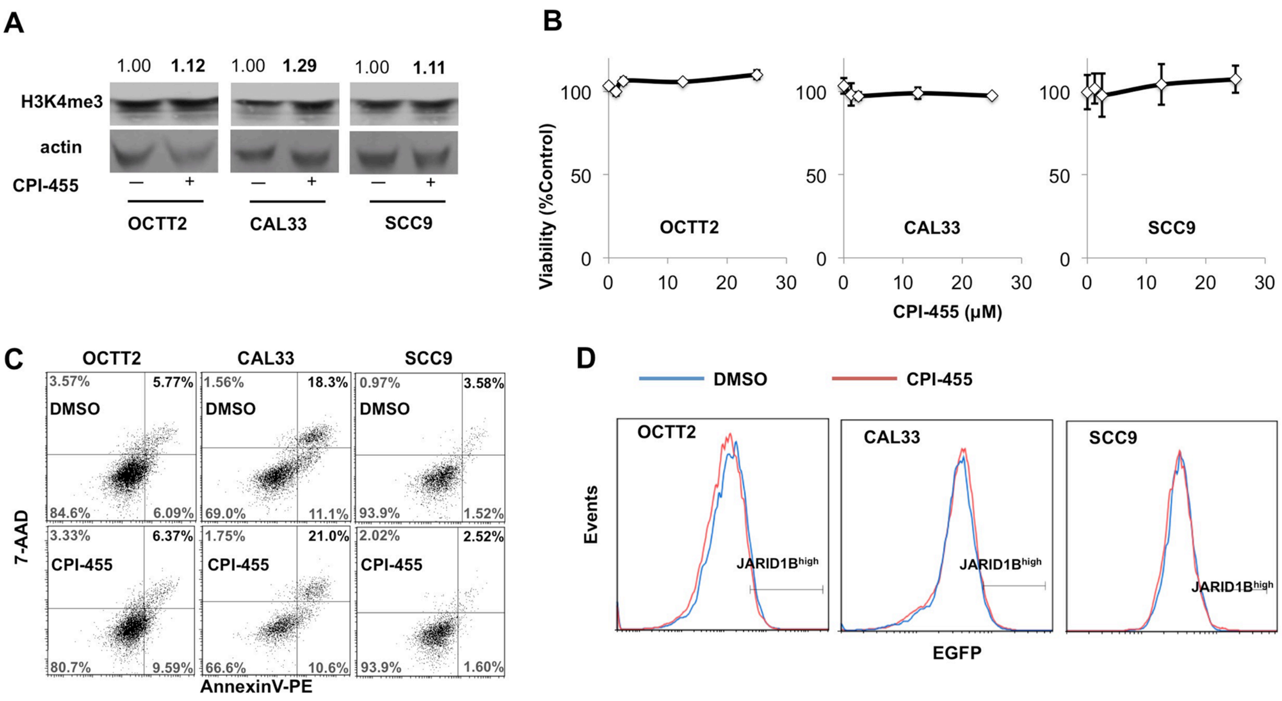

Supplementary Figure 1: CPI-455 has no effect on OSCC cell proliferation or apoptosis. (**A**) WB showing H3K4me3 levels in cell lines treated for 48 hours with CPI-455 (20 μM). Values are H3K4me3 band densities normalized to actin. (**B**) MTS assay of cell lines treated with CPI-455 for 72 hours. (**C**) Apoptotic cells (Annexin-V+/7-AAD+) after CPI-455 (20 μM) treatment. (**D**) JARID1B expression based on EGFP intensity in J1BpromEGFP reporter-containing OSCC cell lines treated for 5 days with CPI-455 (20 μM) or DMSO.

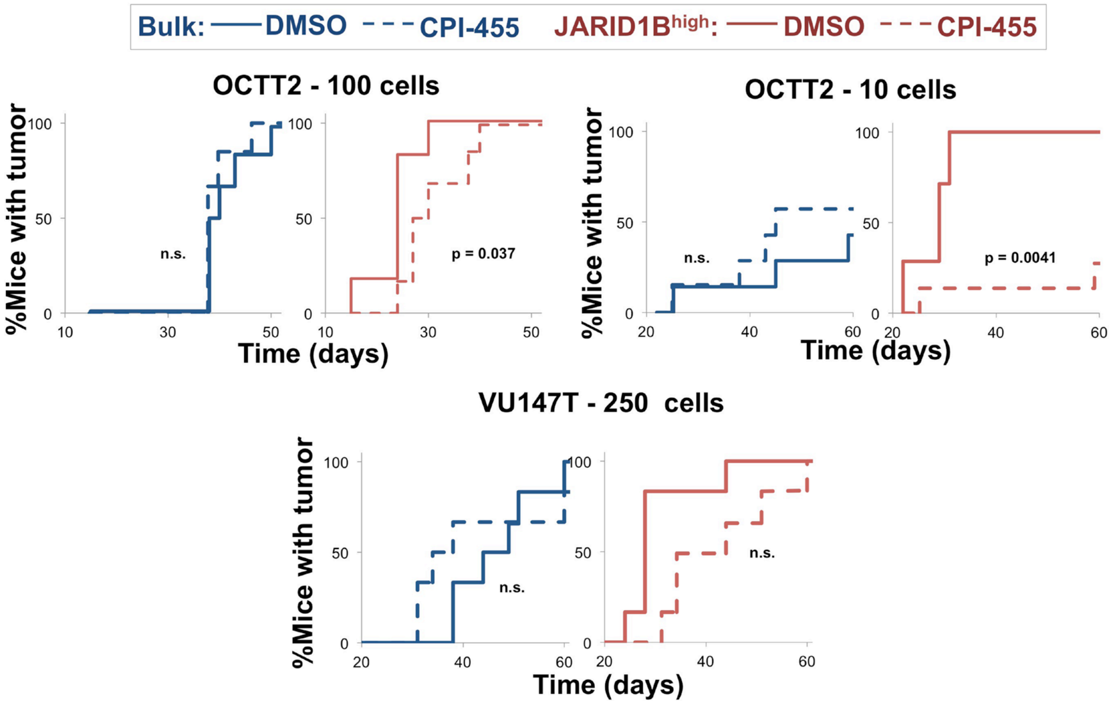

Supplementary Figure 2: CPI-455 pretreatment lengthens tumor latency by JARID1Bhigh but not bulk OSCC cells. Xenograft formation by JARID1B^{high} vs. bulk OCTT2 (left: 100 cells/mouse, n = 6, middle: 10 cells/mouse, n = 7) or VU147T (right: 250 cells/mouse, n = 6) cells pre-treated for 7 days with CPI-455 (20 µM, blue) or DMSO (red). p-values are unadjusted.

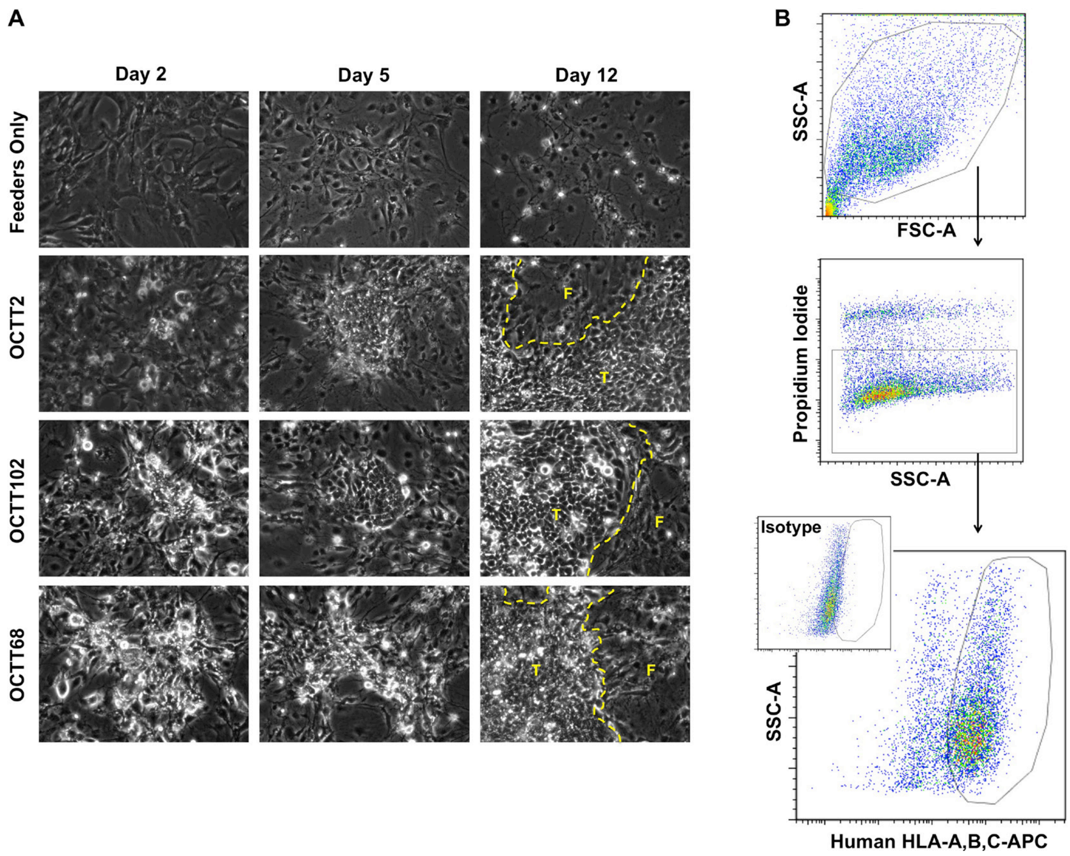

Supplementary Figure 3: Growth and identification of OSCC PDX tumor cells in primary, feeder-assisted cultures. (**A**) Phase contrast images (10×) of feeder-assisted, primary PDX cultures 2, 5, and 12 days post-plating. F = fibroblasts, T = tumor cells. (**B**) Representative FC plots depicting identification of live, PDX tumor cells ($PI^{negative}$/$humanHLA^{positive}$).

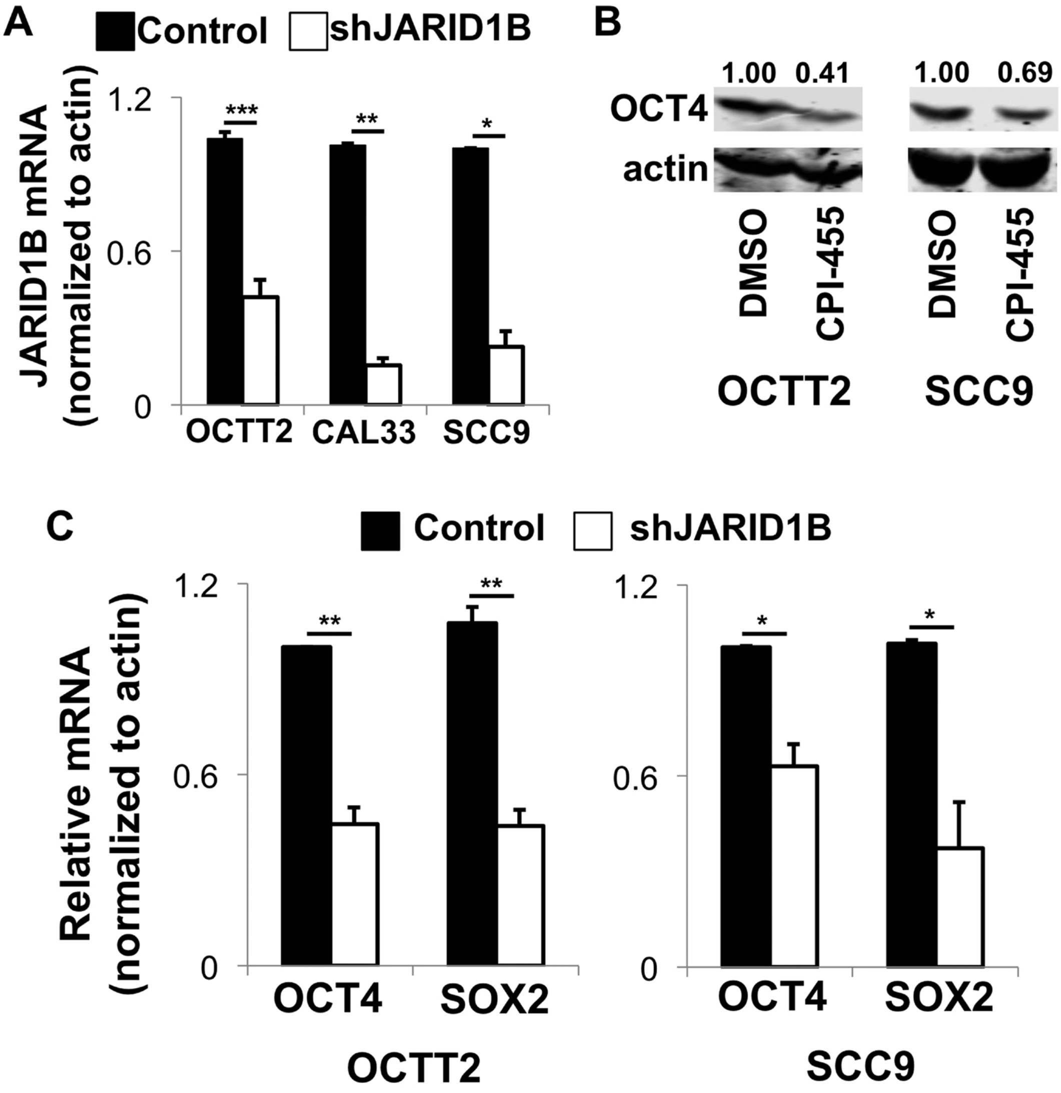

Supplementary Figure 4: Inhibition of JARID1B suppresses OCT4 and SOX expression in OSCC cells. (**A**) JARID1B mRNA by QRT-PCR after lentiviral shRNA knockdown $^{*}p < 0.005$, $^{**}p < 0.001$, $^{***}p < 0.0001$. (**B**) OCT4 protein by WB in OSCC cells treated with CPI-455 (20 μM) or DMSO for 5 days. Values are band densities normalized to actin. (**C**) OCT4 and SOX2 mRNA by QRT-PCR in OSCC cells treated with CPI-455 (20 μM) or DMSO for 5 days $^{*}p < 0.05$, $^{**}p < 0.01$.

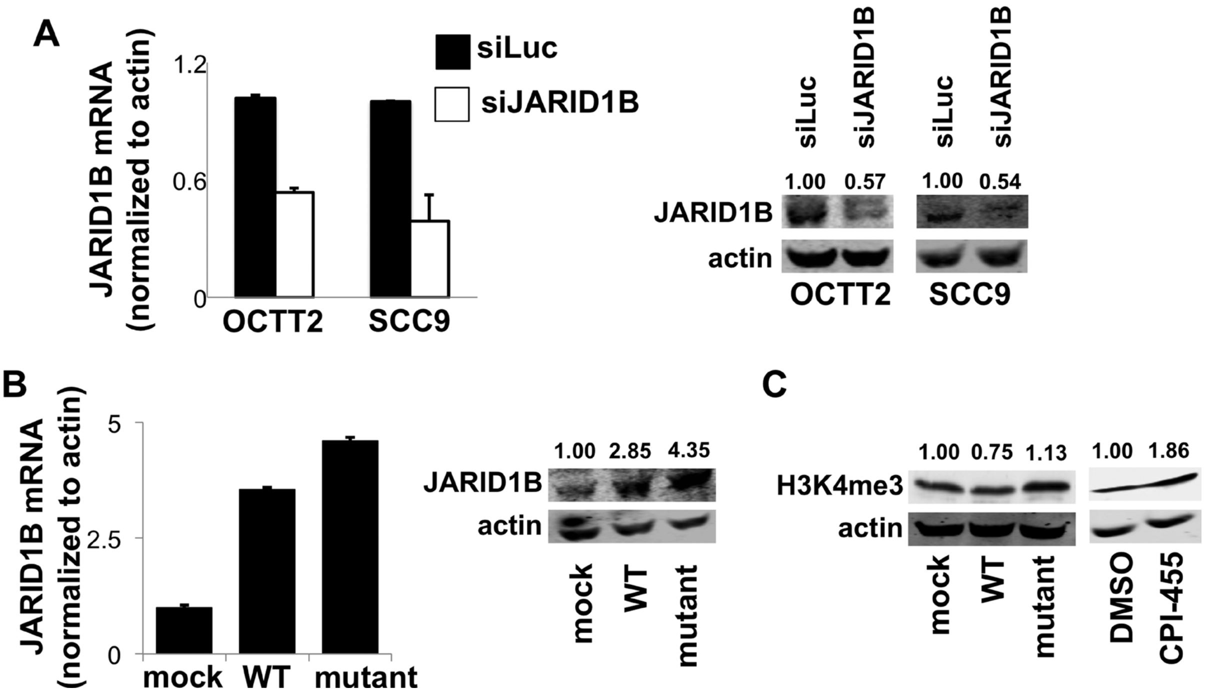

Supplementary Figure 5: Characterization of siRNA-mediated JARID1B silencing and mutant (H499Y) JARID1B overexpression. (**A**) JARID1B mRNA and protein by QRT-PCR and WB, respectively, after transient siRNA knockdown in OSCC cell lines. (**B**) JARID1B mRNA and protein expression in OCTT2 cells overexpressing WT or mutant (H499Y) JARID1B. (**C**) WBs showing side-by-side comparison of H3K4me3 levels in OCTT2 cells overexpressing WT or mutant (H499Y) JARID1B or treated for 5 days with 20 µM CPI-455 (Figure 1A). Values are H3K4me3 band densities normalized to actin.

Supplementary Table 1: List of genes differentially expressed upon stable JARID1B silencing in OCTT2 cells. See Supplementary_Table_1

Supplementary Table 2: List of genes both upregulated in shJARID1B OCTT2 cells and downregulated in JARID1B^{high} OCTT2 cells. See Supplementary_Table_2
